# Flow Control Through the Use of Topography

**DOI:** 10.6028/jres.112.012

**Published:** 2007-06-01

**Authors:** D. L. Cotrell, A. J. Kearsley

**Affiliations:** Lawrence Livermore National Laboratory, Livermore, CA 94550; Mathematical and Computational Sciences Division, National Institute of Standards and Technology, Gaithersburg, MD 20899-8910

**Keywords:** complex boundary, constant properties, finite element method, flow control, fluid dynamics, optimization, steady, Taylor-Couette flow

## Abstract

In this work, optimal shaft shapes for flow in the annular space between a rotating shaft with axially-periodic radius and a fixed coaxial outer circular cylinder, are investigated. Axisymmetric steady flows in this geometry are determined by solving the full Navier-Stokes equations in the actual domain. A measure of the flow field, a weighted convex combination of the volume averaged square of the *L*_2_-norm of the velocity and vorticity vectors, is employed. It has been demonstrated that boundary shape can be used to influence the characteristics of the flow field, such as its velocity component distribution, kinetic energy, or even vorticity. This ability to influence flow fields through boundary shape may be employed to improve microfluidic mixing or, possibly, to minimize shear in biological applications.

## 1. Introduction

Flow control and optimization has been an experimental science for well over 100 years, with an early example being the work by Prandtl [1] in the early 1900s. The bulk of the early work on flow control and optimization fall into two categories: attempts to control fluid motion without the use of sophisticated fluid models involving partial differential equations (e.g., design of heating and cooling systems in a building) and attempts to control fluid motion without the use of sophisticated optimization techniques (e.g., boundary layer control), but using sophisticated fluid models. More recently, because of a better understanding of the equations governing fluid flow, more robust and better optimization algorithms have been employed, together with sophisticated fluid models to solve very complicated flow control and flow design problems. (see for example [2–3]).

Flow in or through a circular annulus driven by rotation of the inner cylinder has been of interest since the work of Taylor [4]. One way to influence this flow is to change the geometry of either the inner or outer cylinder. Rotation of a shaft with axially-varying radius gives rise to a radial velocity component at any non-zero rotation rate, which in turn provides convective mixing in the radial direction. It has been shown that for the case of axisymmetric, axially-periodic variation of the inner cylinder radius, one can achieve significant modification of the flow [5]. By contrast, at rotation rates below the onset of Taylor vortices, a constant-radius shaft provides no radial mixing beyond diffusion. This, and the absence of sharp edges, may make this approach attractive for applications where cell damage must be avoided; thus controlling this flow is of interest.

Selecting an objective function to optimize is an application and problem specific challenge. The governing Navier-Stokes equations are highly nonlinear partial differential equations (quadratic in the velocity), making simulation a computationally demanding task. Moreover, provisions must be made for changes in the shape of the boundary. This can be dealt with by using a grid that includes the entire domain (i.e., grid the solid and fluid portions of the domain) and a grid spacing that does not change from one geometry to another [2]. This method could be inefficient in that grid points in the solid portion of the domain would either need to be removed by algebraic manipulation or have their velocity components set to zero. A fictitious domain method could be employed [2].

In simulation-based optimization settings, a computer simulation must be run repeatedly, in order to compute the various quantities needed by the optimization algorithm. The resulting simulation output must then be postprocessed in order to evaluate the cost function. The cost of computing the cost function is small when compared with that of obtaining derivatives for gradient-based methods, but in many cases these derivatives are not accurate, not available, or simply too costly to calculate [6]. Although automatic differentiation tools have been shown to be effective for some types of simulation-based problems, they are not universally applicable [7]. Results of complex calculations may fail to have the level of precision necessary for a reliable finite-difference approximation to the gradient, eliminating Newton or quasi-Newton methods as possible optimization algorithms. In general, features such as adaptive algorithms, stopping tests in iterative schemes inside the simulation, and the inevitable effect of floating point arithmetic are among the culprits that cause smooth problems to appear nonsmooth [6]. For these reasons, and the fact that one can parameterize a large class of shapes with a small number of parameters (in this case no more than five), a direct search method can be employed to efficiently and effectively find optimal shaft shapes.

The paper is organized as follows: the fluid dynamics problem is reviewed in §2, followed by a description of the optimization approach in §3. Results and discussion are presented in §4, followed by conclusions in §5.

## 2. Flow

This section provides a brief review of the formulation and the numerical method used in computing axisymmetric solutions (i.e., solutions that are invariant along the azimuthal coordinate) of the incompressible Navier-Stokes equations for flow between a fixed outer circular cylinder and a rotating coaxial inner shaft with either rectangular or sinusoidal shape.

Work on extended (i.e., periodic or random) radius variation includes experimental work [8–19], while related work on the effect of localized radius variation has been reported in [20–22]. The most detailed of the experimental investigations are those of [8] and [9] (see [5] for a detailed review of previous work). On the computational side, the most detailed calculations are those of [5], in which bifurcation behavior results as a function of flow rate were presented for a radius ratio of $\eta ={\hat{R}}_{i}/R_{0}=0.5$ (where ${\hat{R}}_{i}$ and *R_0_* are defined below) and several values of the radial amplitude.

### 2.1 Formulation

In this section, we define the governing equations without specifying the exact shape of the boundary. In general, the geometry of interest is that of a fixed outer circular cylinder and an inner coaxial rotating shaft with topography. Axisymmetric steady flows in this geometry are determined by solving the full Navier-Stokes equations in the actual domain. The governing equations and boundary conditions are nondimensionalized by scaling the dimensional coordinates *R* and *Z* with the outer radius, *R_0_*, and the axial wavelength, *L*, respectively, while the velocity and pressure are nondimensionalized using $\Omega_{i}{\hat{R}}_{i}$ and $\rho \Omega_{i}^{2}{\hat{R}}_{i}^{2},$ respectively; where *ρ* is the fluid density, *Ω_i_* is the angular velocity of the inner shaft, and ${\hat{R}}_{i}$ is an appropriate measure of the inner radius (e.g., the mean inner shaft radius). Radius ${\hat{R}}_{i}$ will be defined more precisely, later in this section when the shaft cross-sectional shapes are introduced.

In an inertial reference frame, the dimensionless continuity and momentum equations are

$$\frac{\partial \upsilon_{r}}{\partial r}+\frac{\upsilon_{r}}{r}+\beta \frac{\partial \upsilon_{z}}{\partial z}=0$$ (1a)

$$\upsilon_{r}\frac{\partial \upsilon_{r}}{\partial r}+\beta \upsilon_{z}\frac{\partial \upsilon_{r}}{\partial z}-\frac{\upsilon_{\phi }^{2}}{r}=-\frac{\partial \rho }{\partial r}+\frac{1}{Ta}\left(\nabla^{2}\upsilon_{r}-\frac{\upsilon_{r}}{r^{2}}\right)$$ (1b)

$$\upsilon_{r}\frac{\partial \upsilon_{\phi }}{\partial r}+\beta \upsilon_{z}\frac{\partial \upsilon_{\phi }}{\partial z}+\frac{\upsilon_{r}\upsilon_{\phi }}{r}=\frac{1}{Ta}\left(\nabla^{2}\upsilon_{\phi }-\frac{\upsilon_{\phi }}{r^{2}}\right)$$ (1c)

$$\upsilon_{r}\frac{\partial \upsilon_{z}}{\partial r}+\beta \upsilon_{z}\frac{\partial \upsilon_{z}}{\partial z}=\beta \frac{\partial p}{\partial z}+\frac{1}{Ta}\nabla^{2}\upsilon_{z},$$ (1d)

where

$$\nabla^{2}=\frac{1}{r}\frac{\partial }{\partial r}(r\frac{\partial }{\partial r})+\beta^{2}\frac{\partial^{2}}{\partial z},$$ (2)

*β* = *R*_0_/*L* is the length scale ratio, and $Ta=(\Omega_{i}{\hat{R}}_{i}R_{0})/\nu$ is the Taylor number, where *ν* is the kinematic viscosity of the fluid.

We enforce no-slip conditions on the rigid impermeable inner shaft and outer circular cylinder

$$\begin{array}{ccc} \upsilon_{r}(r_{i}(z),z)=0 & \text{and} & \upsilon_{r}(r_{0},z) \end{array}=0$$ (3a,b)

$$\begin{array}{ccc} \upsilon_{z}(r_{i}(z),z)=0 & \text{and} & \upsilon_{z}(r_{0},z) \end{array}=0$$ (3c,d)

$$\begin{array}{ccc} \upsilon_{\phi }(r_{i}(z),z)=r_{i}(z)/\eta & \text{and} & \upsilon_{\phi }(r_{0},z) \end{array}=0,$$ (3e,f)

and the flow is assumed to be axially periodic

v(r,0)=v(r,1) (4a)

$$\frac{\partial v(r,0)}{\partial z}=\frac{\partial v(r,1)}{\partial z}$$ (4b)

p(r,0)=p(r,1). (4c)

**Fig. 1:**
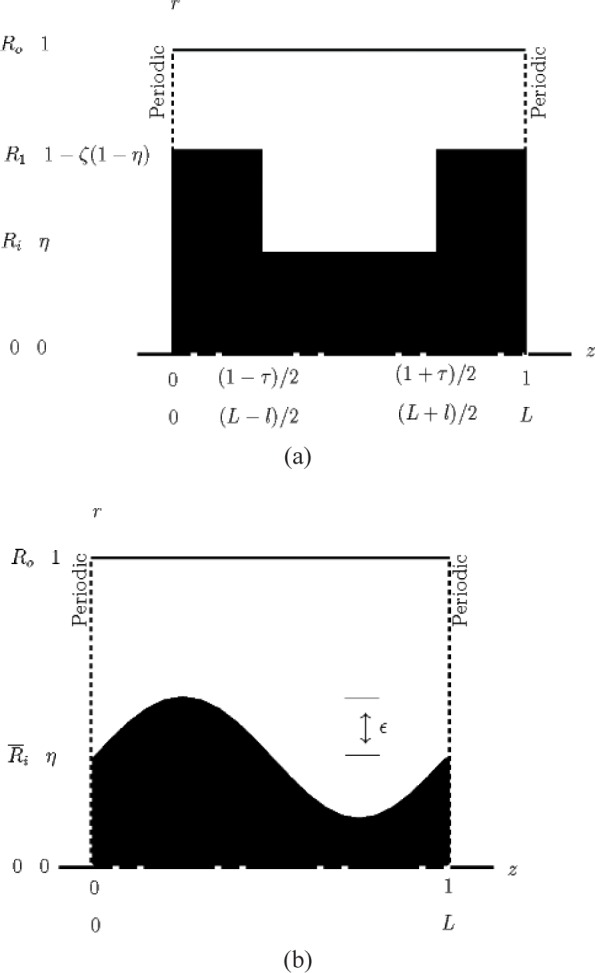
Shaft geometry and computational domain. (a) azimuthal groove of rectangular shape, (b) azimuthal groove of sinusoidal shape.

In this work two shaft shapes are considered; an azimuthal groove of rectangular cross section (Fig. 1a) and an azimuthal groove of sinusoidal cross section (Fig. 1b). In a plane passing through the symmetry axis of the outer cylinder, Figs. 1a and 1b show the two shaft geometries considered. In Fig. 1a, (*ζ* = (*R*_0_ – *R*_l_)/(*R*_0_ – *R*_i_) is the clearance ratio, and *τ* = *l*/*L* is the groove width ratio, where *R*_1_ is the radius of the flight and *l* is the groove width. This geometry is fully defined by setting *β*, *η*, *ζ*, and *τ*. Figure 1b shows the sinusoidal inner shaft geometry with the inner radius varying according to

$$r_{i}(z)=\eta +\varepsilon \sin(2\pi z),$$ (5)

where *ε* is the amplitude of the radial modulation. This geometry is fully defined by setting *β*, *η*, and *ε*.

Note that the precise value of ${\hat{R}}_{i}$ depends on the geometry considered. For the rectangular groove case the radial length scale was chosen to be the hub radius (i.e., ${\hat{R}}_{i}=R_{i}$), while for the sinusoidal groove case the mean inner radius (i.e., ${\hat{R}}_{i}={\bar{R}}_{i}$) is used. Thus, the precise definitions of *η* and *Ta* also depend on which geometry one is considering.

### 2.2 Numerical Method

The momentum and continuity equations are solved numerically using a finite-element method, employing isoparametric quadrilateral elements with quadratic velocity and discontinuous linear pressure interpolation (cf. [23–25]); a consistent penalty method to satisfy incompressibility Eq. (1a) and a 5 × 5 Gaussian quadrature rule for numerical integration. A structured orthogonal mesh with the capability to adjust mesh density throughout the computational domain provides flexibility to locally add more points to resolve relevant flow structures.

Discretization of Eq. (1) leads to a quadratically nonlinear algebraic system of the form

f(v)=0, (6)

which is solved by Newton iteration. If needed, converged solutions at one spatial resolution (or lower *Ta*) can be interpolated to get an initial iterate for the next-higher resolution. The convergence criteria were that both ||**R**|| and ||**v***^n^*^+ 1^ − **v***^n^*|| / ||**v***^n^*|| be less than 10^−4^, where ||⋅|| denotes an *L*_2_-norm [25], **R** is the residual vector used by the Newton iteration, and **v** is the velocity vector. A grid convergence study has been performed for various geometric parameter combinations spanning the ranges considered in the work. Grid spacing gives well converged solutions (i.e., changing less than 1 % with increased spatial resolution) for any parameter combination considered. Results given in this paper are converged to the number of significant figures shown. See [5] for convergence results.

## 3. Optimization

Scalar measures of the flow and an objective function are defined in this section. The problem of finding optimal shapes is considered in two stages; simulation of the fluid dynamics and parameter optimization. A finite element method, as described in the previous section, can be used to perform the simulation computations, while a direct search software package, APPSPACK [26], is employed to estimate optimal flow parameters.

### 3.1 Formulation

Consider the volume averaged *L*_2_-norm squared of the velocity (*γυ*) and vorticity vectors (*γω*)

$$\gamma_{\upsilon }=\frac{\int_{\forall }{\left\|v\right\|}^{2}r\;drdzd\theta }{\int_{\forall }r\;drdzd\theta }$$ (7a)

$$\gamma_{\omega }=\frac{\int_{\forall }{\left\|w\right\|}^{2}r\;drdzd\theta }{\int_{\forall }r\;drdzd\theta }$$ (7b)

where **w** is the vorticity vector. We note that these measures, for example, could be used to investigate mixing using only the velocity field, thus they have not been arbitrarily chosen.

We define the following objective function

$$F={\left[J-J_{d}\right]}^{2}+\frac{\Gamma }{2},$$ (8)

where *J* = *αγ_υ_* + (1 − *α*)*γ_ω_* is a convex combination of Eqs. (7a) and (7b), *α* is a constant taking on values between zero and one, *J_d_* is a desired value of *J*, and *Γ* is a measure of the topography. We note that *Γ* differs between shaft shapes, with it being the dimensionless flight height, (1 − *η*)(1 − *ζ*), for the rectangular groove and the dimensionless amplitude, *ε*, for the sinusoidal groove. Minimumization of Eq. (8) over the geometric parameter space allows one to find the shaft shape that gives a value of *J* closest to the desired value, while also trying to keep the topography to a minimum. We note, for the sinusoidal groove case, that there exists a relationship between *η* and *ε* that must be taken into consideration. This relationship takes the form of a constraint on *ε*

$$0\leq \varepsilon \leq \underset{0\leq \eta \leq 1}{\min}(\eta ,1-\eta ).$$ (9)

Because the software package APPSPACK is employed, which handles only bound constraints, the constraint Eq. (9) must be handled by other means. In this work, constraints are handled through a special penalty function in the same way as employed by [2]. The penalty function penalizes infeasibility but returns a smaller function value for points that simultaneously reduce the objective function and infeasibility. In this way, the algorithm steers iterates away from points that are not physically possible (e.g., the gap width goes negative).

## 4. Results and Discussion

In this work, the simplest axisymmetric flow possible is investigated and optimal shaft shapes are calculated. For a smooth shaft, the location of the first bifurcation from purely azimuthal flow to something more complex (e.g., for *η* = 0.5 the first bifurcation is to a steady and axisymmetric secondary flow and takes place at about *Ta* = 68.19) is strongly dependent on the value of the radius ratio. Significant modifications in solution topology have been shown to take place after the first bifurcation [5]. It is also known that the location of the first bifurcation decreases with increasing radius ratio [27–29]. Previous results [5] suggest that the location of the first bifurcation is significantly affected by topography, where for large amplitude cases results show that the location of the first bifurcation relocates to higher values of *Ta* (i.e., the modified Couette flow is much more stable than the smooth walled case). Computational results for *Ta* = 10 are presented. This value was selected, in part, because it lies below the first bifurcation for any smooth shaft case for the combinations of geometric parameters considered here. This insures that solutions remain on a single solution branch, allowing for the optimal shaft shape to be solution branch independent (see [5] for solution branch details).

In order to gage the effect *β* has on the flow field, we have defined an aspect ratio based on the gap and axial wavelength *Λ* = *H/L*, where *H* = (*R*_0_ − *R_i_*) for the rectangular azimuthal groove case and $H=(R_{0}-{\bar{R}}_{i})$ for the sinusoidal azimuthal groove case. This ratio can also be written in dimensionless form as *Λ* = *β* (1 − *η*), where *η* is defined differently for each shaft shape, as discussed in §3. We note that figures are shown with respect to this aspect ratio.

### 4.1 Rectangular Groove

In this section, optimal shaft shapes for the rectangular groove case are calculated. Geometric control parameters needed to fully define this geometry take on values in the following ranges

$$\begin{array}{ccc} \beta \in [0.5,2.5] & \text{and} & \eta \in [0.2,0.8] \end{array}$$ (10a, b)

$$\begin{array}{ccc} \zeta \in [0.2,0.8] & \text{and} & \tau \in [0.2,0.8] \end{array}.$$ (10c, d)

For each *α*, *F* is minimized for both attainable and unattainable choices of *J_d_*, demonstrating that one can compute an optimal topography that generates this value without changing the rotation speed of the shaft. The results for *α* = 0.5 are qualitatively similar to those for *α* = 0; thus, results for *α* = 0.5 are not shown.

**Fig. 2:**
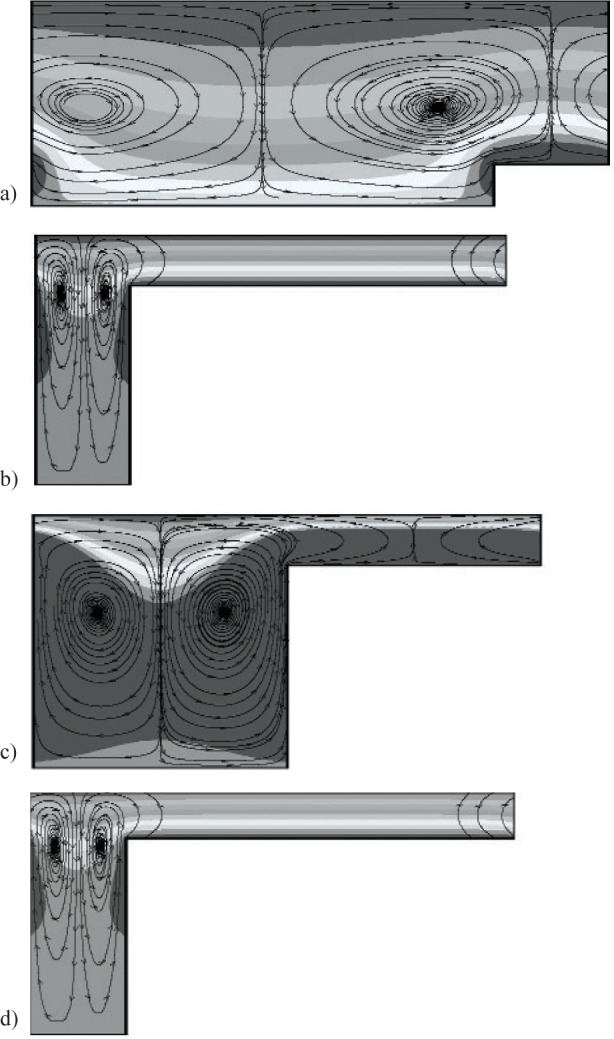
Streamfunction and *υφ* contours for optimal shaft shapes for *Ta* = 10 and *α* = 0. a) Parameter set that minimizes *J*: *β* = 0.5, *η* = 0.29, *ζ* = 0.8, and *τ* = 0.8. b) maximizes *J*: *β* = 2.5, *η* = 0.8, *ζ* = 0.2, and *τ* = 0.2. c) minimizes *F* given an attainable *J_d_*: *β* = 0.59, *η* = 0.2, *ζ* = 0.2, and *τ* = 0.5. d) minimizes *F* given an unattainable *J_d_*: *β* = 2.5, *η* = 0.8, *ζ* = 0.2, and *τ* = 0.2.

***α*** = **0**. For *α* = 0, Figs. 2(a–d) show streamfunction and *υ_ϕ_* (contour values range from 0 to 1.1) contours for the set of geometric parameters that minimize and maximize *J*, and minimize *F*, given an attainable and unattainable *J_d_*, respectively. As seen in Fig. 2a (*Λ* = 0.35) for *β* = 0.5, *η* = 0.294, *ζ* = 0.8, and *τ* = 0.8, minimizing *J* (1.62) leads to a shaft shape with minimal topography and small radius ratio. It is not surprising that the optimal shape would be one of large flight clearance and groove width, given that a measure of the vorticity field is being optimized. In the limit of zero flight height, the flow is unidirectional with only one nonzero vorticity component at this *Ta*. The magnitude of azimuthal shear decreases with decreasing *η*. As seen in Fig. 2b (*Λ* = 0.5) for *β* = 2.5, *η* = 0.8, *ζ* = 0.2, and *τ* = 0.2, maximizing *J* (473) leads again to a shaft shape with minimal topography. In contrast to the results shown in Fig. 2a when minimizing *J*, the topography maximizing *J* has a large radius ratio and small groove width. Because the azimuthal velocity component of the shaft goes as *Ω_i_* R, having a large radius ratio and small-groove width maximizes the magnitude of this component over most of the axial length.

Topography can also fix the measure of the flow field to a desired value. This is of interest, for example, in mixing of biological agents, where excessive shear is known to cause damage. If one knows at what shear rate damage becomes significant, topography control can be used to avoid the maximum shear seen in the field, without changing the rotation rate. As seen in Fig. 2c (*Λ* = 0.48) for *β* = 0.59, *η* = 0.2, *ζ* = 0.2, *τ* = 0.5, and an attainable *J_d_*, minimizing *F* leads to a shaft shape that gives a value of *J* closest to the desired value while limiting changes in the topography to a minimum. For this case, *F* = 0.52 and *Γ* = 0.64. Thus, there is less than a 1 % difference between the *J* (200.45) that minimizes *F* and the desired value (*J_d_* = 200). On the other hand, as shown in Fig. 2d (*Λ* = 0.5), minimizing *F*, given an unattainable value of *J_d_* larger than possible at these rotation rates, leads to a shaft shape (*β* = 2.5, *η* = 0.8, *ζ* = 0.2, *τ* = 0.2) that is identical to that found when maximizing *J*.

**Fig. 3:**
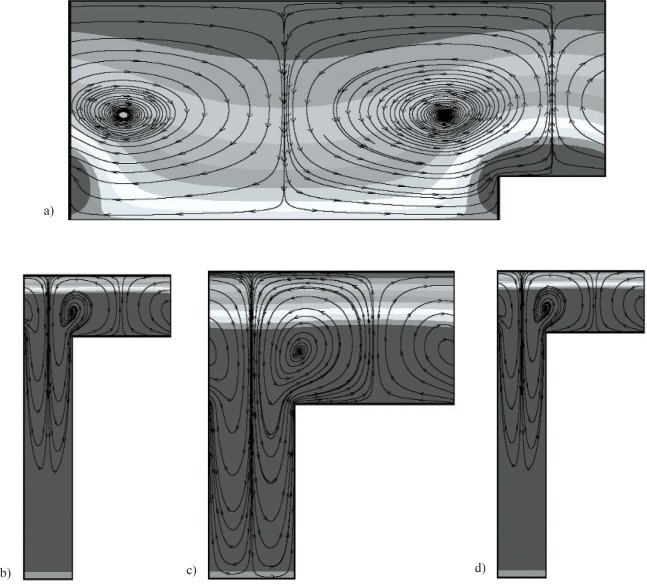
Streamfunction and *υφ* contours for optimal shaft shapes for *Ta* = 10 and *α* = 1. a) Parameter set that minimizes *J*: *β* = 0.5, *η* = 0.2, *ζ* = 0.8, and *τ* = 0.8. b) maximizes *J*: *β* = 2.5, *η* = 0.2, *ζ* = 0.2, and *τ* = 0.33. c) minimizes *F* given an attainable *J_d_* : *β* = 1.5, *η* = 0.2, *ζ* = 0.43, and *τ* = 0.35. d) minimizes *F* given an unattainable *J_d_* : *β* = 2.5, *η* = 0.2, *ζ* = 0.2, and *τ* = 0.33.

***α*** = **1**. For *α* = 1, Figs. 3(a–d) show streamfunction and *υ_ϕ_* (contour values range from 0 to 1.1, with there being ten divisions) contours for the set of geometric parameters that minimize and maximize *J*, and minimize *F*, given an attainable and unattainable *J_d_*, respectively. As seen in Fig. 3a (*Λ* = 0.4) for *β* = 0.5, *η* = 0.2, *ζ* = 0.8, and *τ* = 0.8, minimizing *J* once more leads to a shaft shape with minimal topography. The optimal shaft shape turns out to be one of large flight clearance and groove width, and small radius ratio. This is consistent with what one would expect when minimizing a measure of the kinetic energy. As seen in Fig. 3b (*Λ* = 2) for *β* = 2.5, *η* = 0.2, *ζ* = 0.2, and *τ* = 0.33, maximizing *J* leads to a shaft shape having significant topography (i.e., a deep thin groove). This differs from the *α* = 0 results, where the radius ratio was large.

As shown in Fig. 3c (*Λ* = 1.2) for *β* = 1.5, *η* = 0.2, *ζ* = 0.43, *τ* = 0.35, and an attainable *J_d_*, minimizing *F* leads to a shaft shape having medium flight clearance and small groove width. For this case, *F* = 0.23 and *Γ* = 0.46. Also, for this case we have used the topography to exactly match the desired value of *J_d_* = 3. We note that this value of *β* is significantly different than the values found for *α* = 0. The results (see Fig. 3d where *Λ* = 2) for an unattainable value of *J_d_* are similar to those for *α* = 0.

### 4.2. Sinusoidal Groove

In this section, optimal shaft shapes for the sinusoidal groove case are calculated. Geometric parameters needed to fully define this geometry are constrained to take on values in the following ranges

$$\begin{array}{ccc} \beta \in [0.5,2.5] & \text{and} & \eta \in [0.2,0.8] \end{array}$$ (11a, b)

ε∈[0,0.5], (11c)

where the upper bound on *ε* is taken to be 1/2 of the maximum value of the right-hand-side of Eq. (9). A minimum gap between the inner and outer cylinders of 0.05 is enforced. This is required for numerical reasons, because numerically it is desirable that a sufficient number of elements are present inside the gap to yield accurate solutions. The results for *α* = 0.5 are again qualitatively similar to those for *α* = 0, as was the case for the rectangular groove case, and again are not shown.

**Fig. 4:**
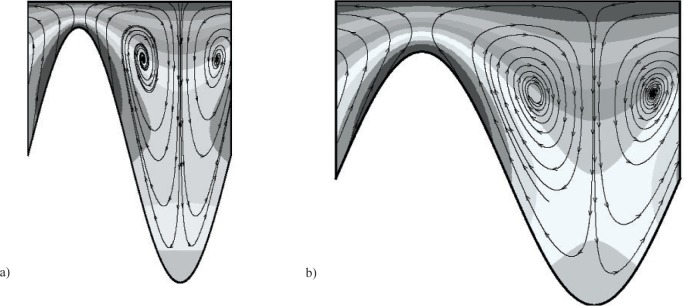
Streamfunction and *υ_ϕ_* contours for optimal shaft shapes for *Ta* = 10 and *α* = 0. a) Parameter set that maximizes *J*: *β* = 2.5, *η* = 0.70, and *ε* = 0.25. b) minimizes *F* given an attainable *J_d_* : *β* = 1.25, *η* = 0.65, and *ε* = 0.25.

***α*** = **0**. For *α* = 0, Figs. 4(a–b) show streamfunction and *υ_ϕ_* (contour values range from 0 to 1.1, with there being ten divisions) contours for the set of geometric parameters that maximize *J* and minimize *F* given an attainable *J_d_*, respectively. The optimal shaft shape that minimizes *J* has no topography. In the limit *ε* → 0, the flow is unidirectional at this *Ta* and has only one nonzero vorticity component. For any nonzero value of *ε*, there are three nonzero velocity components, and thus all three vorticity components are nonzero. As seen in Fig. 4a for *β* = 2.5, *η* = 0.70, and *ε* = 0.25, maximizing *J* leads to a shaft shape with significant topography. With *Λ* = 0.76, the mean gap is smaller than the axial wavelength. We note that as *β* increases while maintaining a constant mean gap width, the gradients in the flow also increase. This, along with the fact that *w_r_* scales with *β*, causes *γ_w_* to increase as well. This optimal shaft shape is very similar to those found for the rectangular groove case, with *β* being the same, *η* about 12 % less, and the minimum gap width chosen in each case. In contrast, the overall aspect ratio (*Λ*) is very different for these two cases.

As seen in Fig. 4b for *β* = 1.25, *η* = 0.65, *ε* = 0.25, and an attainable *J_d_*, minimizing *F* leads to a shaft shape that gives a value of *J* closest to the desired value, while keeping the topography to a minimum. For this case, *F* = 0.17 and *Γ* = 0.25. As was the case for all the rectangular groove cases, there is less than a 1 % difference between the *J* (25.09) that minimizes *F* and the desired value (*J_d_* = 25). On the other hand, as was also the case for the rectangular groove case, minimizing *F*, given an unattainable value of *J_d_* leads to a shaft shape (*β* = 2.5, *η* = 0.70, and *ε* = 0.25), identical to that found when maximizing *J*.

**Fig. 5:**
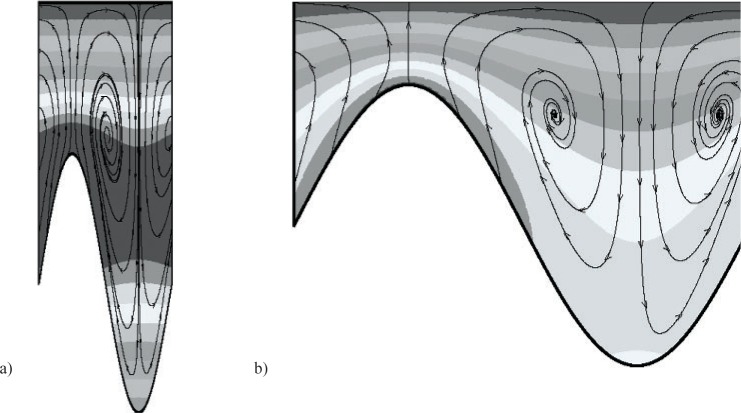
Streamfunction and *υ_ϕ_* contours for optimal shaft shapes for *Ta* = 10 and *α* = 1. a) Parameter set that maximizes *J*: *β* = 2.5, *η* = 0.35, and *ε* = 0.30. b) minimizes *F* given an attainable *J_d_* : *β* = 2.5, *η* = 0.8, and *ε* = 0.125.

***α*** = **1**. For *α* = 1, Figs. 5(a–b) show streamfunction and *υ_ϕ_* (contour values range from 0 to 1.1, with there being ten divisions) contours for the set of geometric parameters that maximize *J* and minimize *F*, given an attainable *J_d_*, respectively. As was the case for *α* = 0, the shaft shape that minimizes *J* has no topography. As seen in Fig. 5a for *β* = 2.5, *η* = 0.35, and *ε* = 0.30, maximizing *J* leads to a shaft shape with moderate topography compared to the *α* = 0 case. As seen in Fig. 5a, *Λ* = 1.63 for this case, thus the mean gap width is much larger than the wavelength.

As seen in Fig. 5b for *β* = 2.5, *η* = 0.8, *ε* = 0.125, and an attainable *J_d_*, minimizing *F* leads to a shaft shape that gives a value of *J* closest to the desired value, while again keeping the topography to a minimum. For this case, *F* = 0.08 and *Γ* = 0.125. There is about a 39 % difference between the *J* (0.98) that minimizes *F* and a desired value (*J_d_* = 0.6). This value is significantly larger than the value found for *α* = 0 and any of the rectangular groove cases. For *α* = 1, the range of *J* is much smaller than that found for *α* = 0, making it more difficult to find the topography that gives the desired value. On the other hand, as was also the case for *α* = 0 and all the rectangular groove cases, minimizing *F*, given an unattainable value of *J_d_* leads to a shaft shape (*β* = 2.5, *η* = 0.35, and *ε* = 0.30) identical to that found when maximizing *J*.

## 5. Conclusions

It has been demonstrated that boundary shape can be used to influence the characteristics of the flow field, such as its velocity component distribution, kinetic energy, or even vorticity. The geometric parameters investigated in this work are those associated with mixing machinery and instruments being developed and used today. The work here provides a first step in designing an optimization tool for designing experiment and operation specific fluid mixing instruments. This ability to influence flow fields through boundary shape may be employed to improve microfludic mixing, maximize through flow while trying to improve low speed mixing, or possibly to minimize shear in biological applications (e.g., separations or downstream of artificial heart valves).
